# Sensitivity-controllable refractive index sensor based on reflective θ-shaped microfiber resonator cooperated with Vernier effect

**DOI:** 10.1038/s41598-017-10163-x

**Published:** 2017-08-29

**Authors:** Zhilin Xu, Yiyang Luo, Deming Liu, Perry Ping Shum, Qizhen Sun

**Affiliations:** 1 School of Optical and Electronic Information, National Engineering Laboratory for Next Generation Internet Access System, Huazhong University of Science and Technology, Wuhan, 430074 Hubei China; 2 CINTRA CNRS/NTU/THALES, Singapore, 637553 Singapore; 3 School of Electrical and Electronic Engineering, Nanyang Technological University, 639798 Singapore, Singapore

## Abstract

In this paper, we report a sensitivity-controllable refractive index (RI) sensor based on a reflective θ-shaped microfiber resonator cooperated with Vernier effect. The θ-shaped microfiber resonator is a reflective all-fiber device with comb spectrum under weak coupling condition. By cascading it with a fiber Fabry-Perot interferometer, Vernier effect is generated to demodulate surrounding RI with enhanced sensitivity. Theoretical analysis reveals that RI sensitivity of the combined structure with Vernier effect is *m* times higher than the sensitivity of singular θ-shaped microfiber resonator. Moreover, by adjusting cavity length of the θ-shaped microfiber resonator, magnification factor *M* = (*m* + 1) can be tuned which enables the RI sensitivity to be controlled. Experimental result demonstrates that the RI sensitivity can be widely tuned from 311.77 nm/RIU (Reflective index unit) to 2460.07 nm/RIU when the cavity length of the θ-shaped microfiber resonator is adjusted from 9.4 mm to 8.7 mm. The θ-shaped microfiber resonator based all-fiber RI sensor featuring controllable sensitivity and compact size can be widely used for chemical and biological detections. The proposed scheme of generating Vernier effect also offers a universal idea to increase measurement sensitivity for optical fiber sensing structures with comb spectrum.

## Introduction

Refractive index (RI) of liquid is a basic quantity that can be applied to characterize the changes of many chemical and biological measurands, such as gas concentration^1^, seawater salinity^2^, PH values^3^, DNA^4^, glucose concentration in serum^5^, *etc*. Resulting from the important roles in the chemical and biological fields, developing sensitive optical RI detectors is of fundamental interest and great significance^6–8^. Benefiting from the unique advantages of thin diameter and large evanescent field, microfiber is believed to be an excellent medium for constructing miniature RI sensors^9, 10^. Indeed, abundant microfiber refractometers were developed in the past years. Microfiber gratings once attracted considerable attention, due to their narrow bandwidth, small size and good measurement accuracy. But the complicated fabrication processes, fragile structures and rough packaging limit their practical applications^11–15^. Microfiber in-line interferometers such as modal interferometers and Fabry-Perot interferometers (FPIs) were also investigated for RI sensing^16–19^. As illustration, Wen Bin Jin *et al*. utilized a non-adiabatic microfiber assisted by facet reflection of a patch cord to realize an RI sensor with sensitivity high to 18681.82 nm/RIU (refractive index unit)^18^. Nevertheless, these sensing structures encounter problems of uncontrollable fabricating process and poor re-configurability. Moreover, due to the features of high Q-factor, further-reduced size, and easy fabrication, much effort has been dedicated to RI sensors based on microfiber resonators^20–22^. For instance, an embedded microfiber coil resonator was studied for RI detection with resolution as low as 10^−10^, whereas its sensitivity is only 700 nm/RIU even when the microfiber diameter is down to 300 nm^20^. Aside from the short sensitivity, the existing resonators always rely on additional fiber tapers to lead transmission signal out, which further makes the microfiber resonator systems fragile and lossy. A reflective microfiber resonator can cover the shortage but has not been reported yet.

As an efficient way to enhance measurement sensitivity, Vernier effect has attracted considerable attention from the optical sensing field. Vernier effect induced by two cascaded silicon micro-ring resonator has been abundantly studied for RI detection^23–25^. But the high cost and incompatibility with fiber devices are obstacles to their applications in the popular fiber systems. As for the fiber based Vernier effect, cascaded fiber ring resonators^26^ and cascaded intrinsic FPIs^27^ are demonstrated the ability of effectively measuring strain and magnetic field, respectively. Nevertheless, they are unable to measure RI due to lack of effective interaction with analyte. Furthermore, the existing structures with Venier effect are usually composed of two identical elements^23–27^, one as sensing element and the other as reference element. However, in these situations, the reference elements usually cannot fully isolate from the measurand and thus will disturb the sensing results.

In this paper, we propose a sensitivity-controllable RI probe based on a robust θ-shaped microfiber resonator cooperated with Vernier effect. The θ-shaped microfiber resonator is a specially designed all-fiber reflective device with comb spectrum under weak coupling condition. By cascading the θ-shaped microfiber resonator with a fiber Fabry-Perot interferometer (FFPI), Vernier effect is generated to demodulate surrounding RI variation with enhanced sensitivity. Both theoretical analyses and experimental demonstration are conducted to investigate the sensing performances of the proposed RI sensor.

## Results

### Optical and RI sensing characteristics of the θ-shaped microfiber resonator

**Figure 1:**
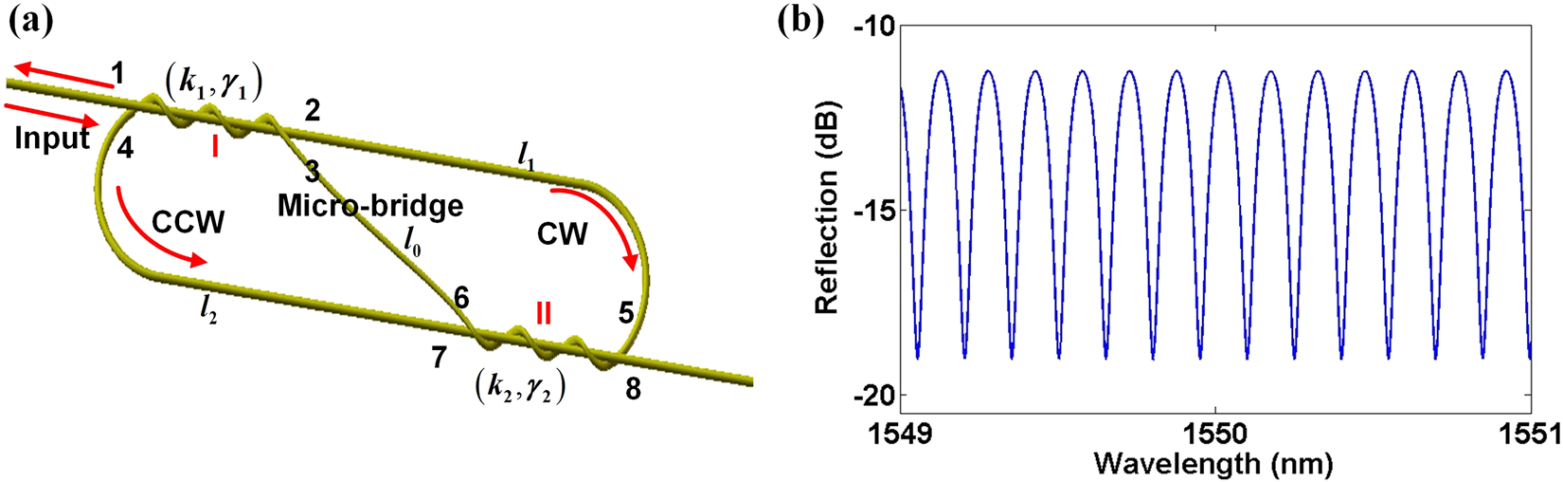
(**a**) Schematic of the θ-shaped microfiber resonator. (**b**) A typical simulated reflection spectrum of the θ-shaped microfiber resonator with *κ* = 0.1 and *γ* = 0.5.

Schematic of the θ-shaped microfiber resonator is shown in Fig. 1, where an elliptical cavity is divided by a “micro-bridge” into shape of character “θ”. In the structure, coupling efficiencies and coupling losses of two couplers I, II are (*κ*_1_, *γ*_1_) and (*κ*_2_, *γ*_2_) respectively, as marked in Fig. 1(a). In this work, *κ*_1_ = *κ*_2_ = *κ* and *γ*_1_ = *γ*_2_ = *γ* are taken for simplification, since the two couplers are symmetric for the resonator. Referring to Fig. 1(a), light is launched into the cavity through port 1, and clockwise (CW) mode oscillation along path of 2-5-7-4-2-5-7-……is initially generated. In the propagation process, the CW mode arriving at the coupler I or the coupler II will be divided into two branches. One branch straightly passing through the coupler maintains oscillation as the CW mode, while the other branch being routed to the micro-bridge is converted into a counter clockwise (CCW) mode along the path of 4-7-5-2-4-7-5-……A part of CCW signal can be output as reflection through port 1, as indicated in Fig. 1(a). By using coupled mode theory, reflectance of the resonator can be derived as:

1 $$\begin{array}{rcl}{\rm{r}} & = & {\rm{j}}\cdot \sqrt{{(1-\gamma )}^{2}(1-\kappa )\kappa }\exp [j\beta ({l}_{1}+{l}_{0})]\\  &  & \times \{2\cdot \sqrt{(1-\gamma )(1-\kappa )}\frac{(1-\gamma )(1-\kappa )\exp (j\beta L)}{{[1+(1-\gamma )\kappa \exp (j\beta L)]}^{2}}+\frac{2\sqrt{(1-\gamma )(1-\kappa )}}{1+(1-\gamma )\kappa \exp (j\beta L)}\}\end{array}$$

Here, *β* is the propagation constant of resonant modes in the θ-shaped microfiber resonator. *l*_0_, *l*_1_ and *l*_2_ are the distances of micro-bridge (port 3-port 6), upper arm (port 2-port 5), lower arm (port 7-port 4), respectively. *L* = *l*_1_ + *l*_2_ is the length of main cavity. Then the reflection amplitude can be calculated by ${\rm{R}}={|r|}^{2}$. It can be inferred from Eq. (1) that under weak coupling condition, namely the coupling coefficient *κ* is relatively small (*κ* < 0.2) and coupling loss *γ* is relatively high (*γ* > 0.1), the reflective θ-shaped microfiber resonator is comb spectrum. A typical reflection spectrum of the θ-shaped microfiber resonator with *κ* = 0.1 and *γ* = 0.5 is shown in Fig. 1(b).

Equation (1) also indicates that the reflection spectrum of the θ-shaped microfiber resonator is partly determined by the propagation constant *β*. Since light in the θ-shaped microfiber resonator is guided as evanescent wave, change of the RI in surrounding medium will result in the change of the propagation constant *β*, causing the resonant wavelength in the reflection spectrum to be shifted. Assuming that the surrounding RI *n*_*a*_ is varied by Δ*n*_*a*_, the resonant wavelength *λ*_1_ will be changed by:

2 $$\Delta {\lambda }_{1}={\lambda }_{1}(\Delta {n}_{a}/{n}_{eff1})(\Delta {n}_{eff1}/\Delta {n}_{a})$$

Then, RI sensitivity of the θ-shaped microfiber resonator can be expressed as:

3 $${S}_{1}=\Delta {\lambda }_{1}/\Delta {n}_{a}={\lambda }_{1}/{n}_{eff1}\cdot \Delta {n}_{eff1}/\Delta {n}_{a}$$

where *n*_*eff*1_ and Δ*n*_*eff*1_ are the original effective RI of modes in θ-shaped microfiber resonator and its variation. Δ*n*_*eff*1_/Δ*n*_*a*_ means the rate of change of effective mode RI to the change of surrounding RI, which is determined by the microfiber diameter. *λ*_1_is the operating wavelength. By applying the parameters in Table 1 into Eqs (1–3), we calculate the RI sensitivity of the θ-shaped microfiber to be 24.58 nm/RIU, which, however, is ultra-limited for most chemical and biological applications. Although decreasing the microfiber diameter might increase the RI sensitivity due to a larger value of Δ*n*_*eff*1_/Δ*n*_*a*_^28^, the increment would still be too little to be counted on. Therefore, another effective way to enhance the RI sensitivity is highly desired.

**Table 1:** Simulation parameters for predicting RI sensing characteristics of the θ-shaped microfiber resonator.

Coupling efficiency *κ*	Coupling loss *γ*	Microfiber diameter (μm)	Cavity length *L* (mm)	Original RI *n*_*a*_
0.1	0.01	2	4	1.3320

### Vernier effect between θ-shaped microfiber resonator and FFPI

**Figure 2:**
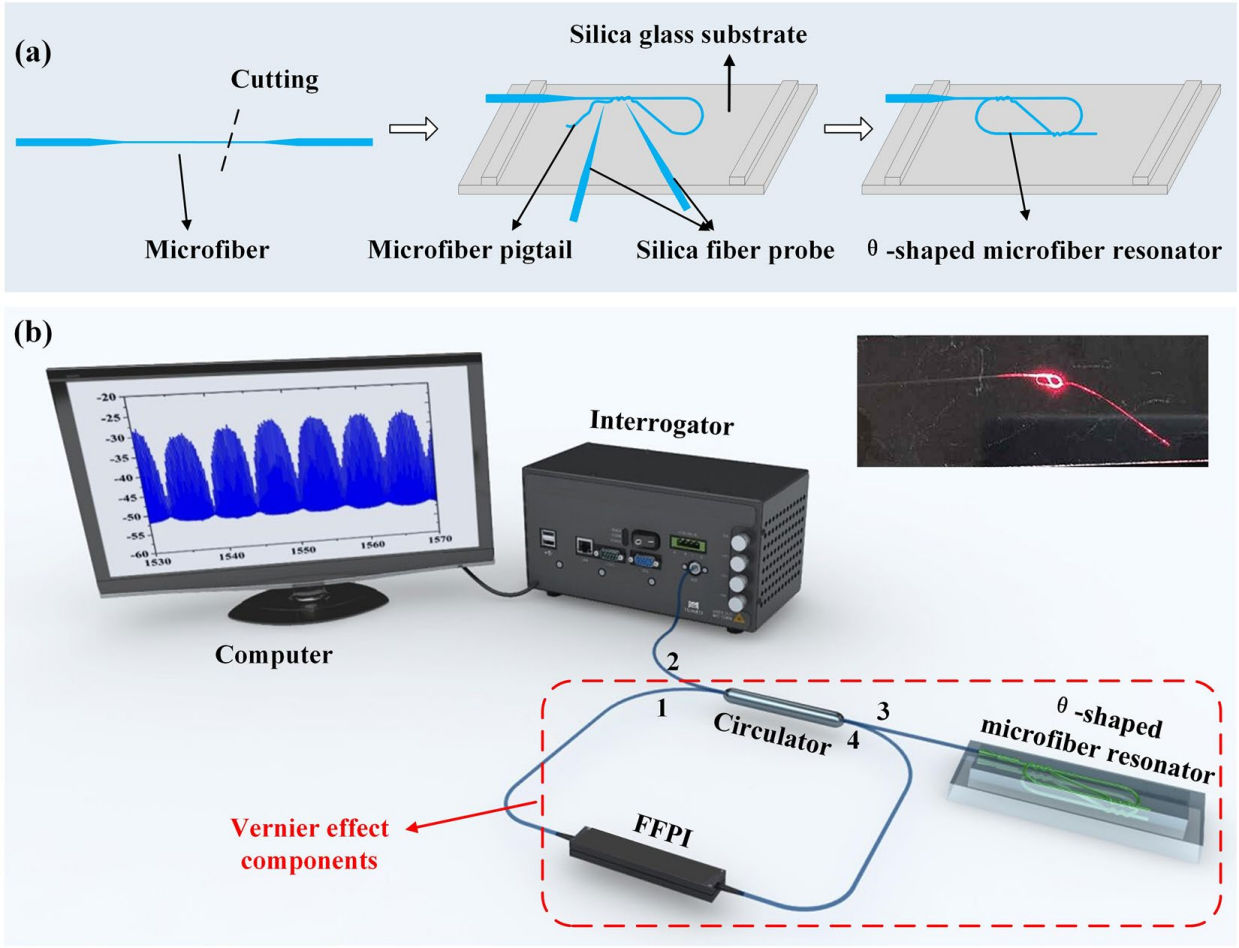
(**a**) Schematic of fabrication process of the θ-shaped microfiber resonator. (**b**) Experimental setup for RI sensing characterization of the θ-shaped microfiber resonator. (Inset: an experimentally fabricated θ-shaped microfiber resonator.) FFPI: fiber Fabry-Perot interferometer.

Considering its ability of effectively enhancing measurement sensitivity, Vernier effect occurs to us as a potential method to increase the RI sensitivity. Since the refection spectrum of the θ-shaped microfiber resonator is comb spectrum, another comb spectrum should be employed to induce Vernier effect. As an optically stable and commercially accessible fiber device, FFPI is definitely a good candidate for achieving the other comb spectrum. Based on the above analysis, the θ-shaped microfiber resonator is cascaded with a FFPI to generate Vernier effect, as shown in Fig. 2(b). This also offers a universal idea of generating Vernier effect to increase measurement sensitivity for other fiber sensing structures with comb spectra.

**Figure 3:**
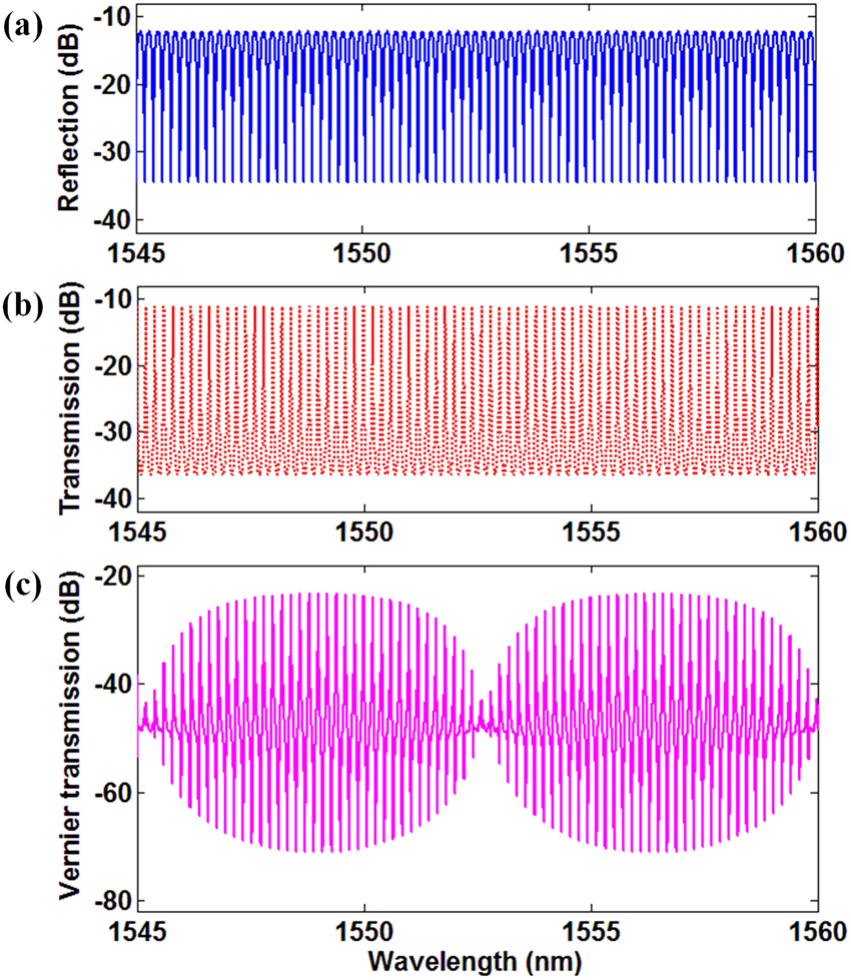
Principle of Vernier effect. (**a**) Reflection spectrum of the θ-shaped microfiber resonator; (**b**) Transmission spectrum of the FFPI; (**c**) Transmission spectrum of the combined structure with Vernier effect.

In order to generate Vernier effect, free spectrum ranges (FSRs) of the θ-shaped microfiber resonator (*FSR*_1_) and the FFPI (*FSR*_2_) should meet the rule of $FS{R}_{1}/FS{R}_{2}=m/(m+1)\,(m=1,\,2,\,3,\ldots )$. Then, periodicity of the resulting Vernier spectrum (*FSR*_*r*_) can be deduced as^23–27^:

4 $$FS{R}_{r}=(m+1)\times FS{R}_{1}=m\times FS{R}_{2}$$

where *FSR*_1_ can be obtained by $FS{R}_{1}={\lambda }^{2}/{n}_{eff1}L$, and *FSR*_2_ can be chosen from the commercial FFPI. It is obvious that *FSR*_*r*_ is *m* times higher than *FSR*_1_, as illustrated in Fig. 3. Due to the spectrum magnification function of the Vernier effect, the RI sensitivity of the θ-shaped microfiber resonator after cascading with the FFPI will be magnified, and can be inferred as^23^:

5 $$S=[FS{R}_{2}/(FS{R}_{2}-FS{R}_{1})]\cdot ({\lambda }_{1}/{n}_{eff1})\cdot (\Delta {n}_{eff1}/\Delta {n}_{a})=(m+1)\cdot {S}_{1}$$

It is found from Eqs (3) and (5) that compared with the sensitivity of singular θ-shaped microfiber resonator, the sensitivity after combining with a FFPI is enhanced by $M=[FS{R}_{2}/(FS{R}_{2}-FS{R}_{1})]=(m+1)$, because of which *M* is defined as the magnification factor. Additionally, since *FSR*_1_ can be tuned by adjusting the cavity length of the θ-shaped microfiber resonator, the *M* can also be tuned, enabling the RI sensitivity of the combined structure to be controllable. The RI sensitivity-controllability of the θ-shaped microfiber resonator endorses it to meet different requirements of various applications.

### Experimental spectra of the θ-shaped microfiber resonator and Vernier effect

**Figure 4:**
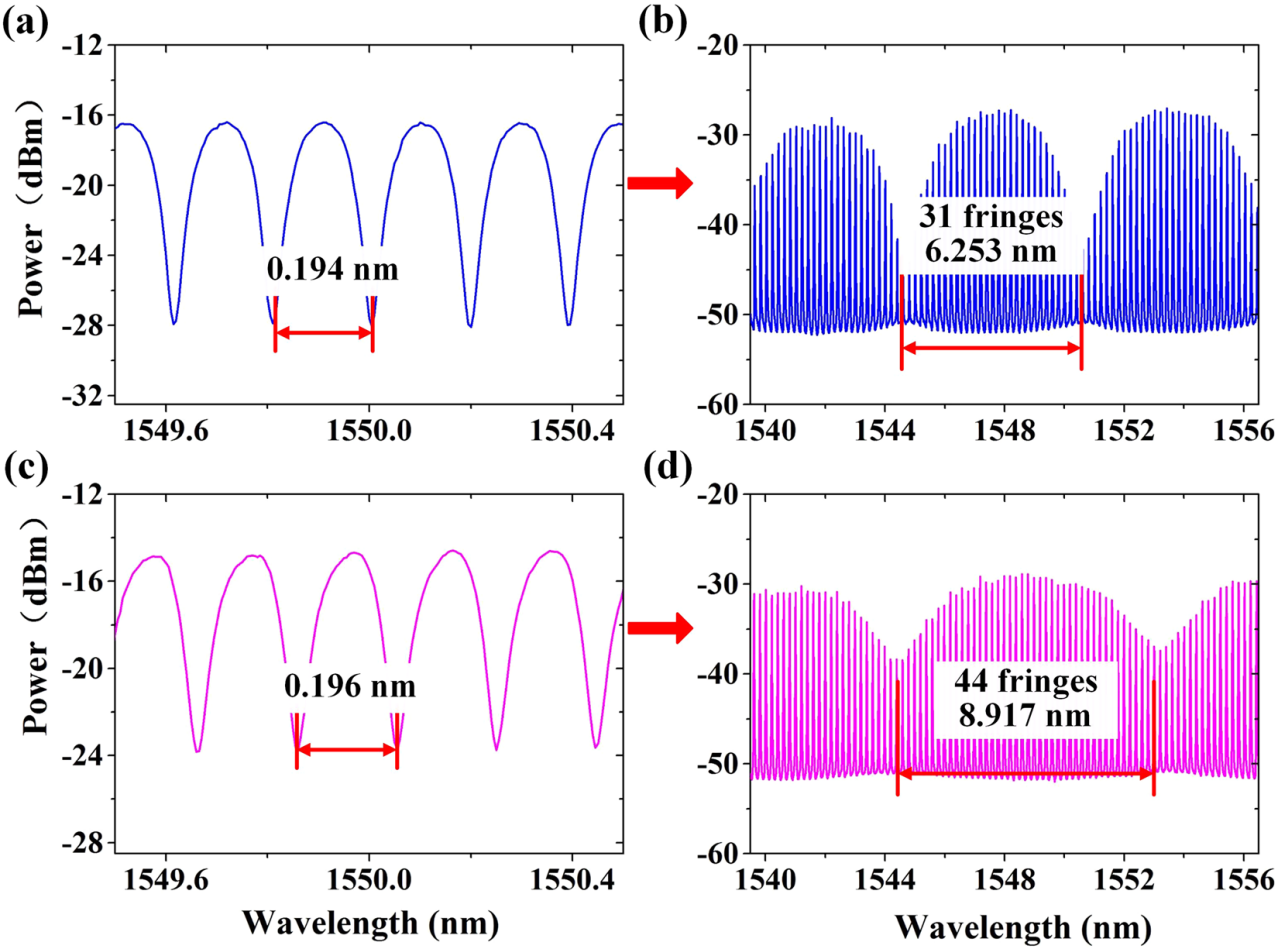
Typical experimental spectra and tunability of Vernier spectrum. (**a**) and (**b**) are reflection spectrum of the θ-shaped microfiber resonator with *FSR*_1_ = 0.194 *nm* and the corresponded Vernier spectrum with *FSR*_*r*_ = 6.253 *nm*, respectively. (**c**) and (**d**) are reflection spectrum of the θ-shaped microfiber resonator *FSR*_1_ = 0.196 *nm* and the corresponded Vernier spectrum with *FSR*_*r*_ = 8.917 *nm*, respectively.

FSR of the FFPI (*FSR*_2_) utilized in this paper is 0.2 nm. Therefore, the FSR of the θ-shaped microfiber resonator (*FSR*_1_) should be controlled approaching 0.2 nm in accordance with the rule in Eq. (4). An experimentally measured reflection spectrum of the θ-shaped microfiber resonator and the induced Vernier spectrum are shown in Fig. 4(a) and (b), where the FSRs are 0.194 nm and 6.253 nm, respectively. About 31 fringes exist within a Vernier period, corresponding to a Vernier effect induced magnification factor of *M* = 31. Then, we changed the *FSR*_1_ into 0.196 nm by adjusting the cavity length of θ-shaped microfiber resonator as shown in Fig. 4(c). *FSR*_*r*_ was changed accordingly to 8.917 nm, as illustrated in Fig. 4(d), which demonstrates the tunablity of the Vernier spectrum.

### Basic RI detection performance

**Figure 5:**
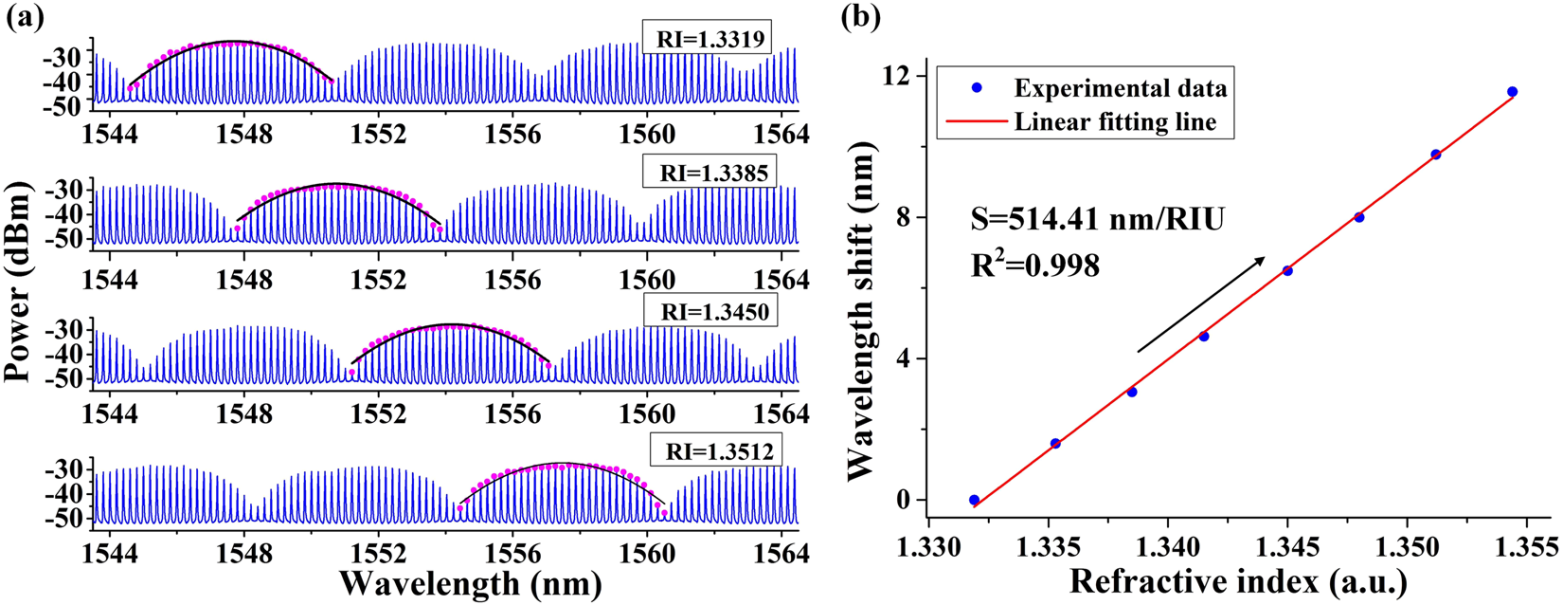
(**a**) Transmission spectra under different surrounding RIs. In order to accurately locate the Vernier peaks, Lorentz fitting algorithm is applied to a chosen period of the Vernier spectra. The blue lines are measured Vernier spectra; the purple points are peaks of the sub-fringes; the red lines are Lorentz fitting curves. (**b**) Wavelength shift of the Vernier peak as a function of surrounding RI.

In the experiment, the surrounding RI was changed from 1.3319 to 1.3550 by using glycerin solution with different concentration. Transmission spectra under different surrounding RIs are presented in Fig. 5(a). To accurately locate the peak of Vernier envelope and continuously measure the RI changing, Lorentz fitting algorithm is applied to a chosen period of the Vernier spectra, as illustrated by black lines in Fig. 5(a) ^25^. The center of the Lorentz fitting curve is regarded as the Vernier peak of the chosen period. It is clearly observed from Fig. 5(a) that the Vernier peak shifts to longer wavelength as the RI increasing. Wavelength shift of the Vernier peak as a function of surrounding RI is displayed in Fig. 5(b). The RI sensitivity is measured to be 514.4 nm/RIU, with linearity of *R* ^2^ = 0.998.

### RI sensitivity controllability

**Figure 6:**
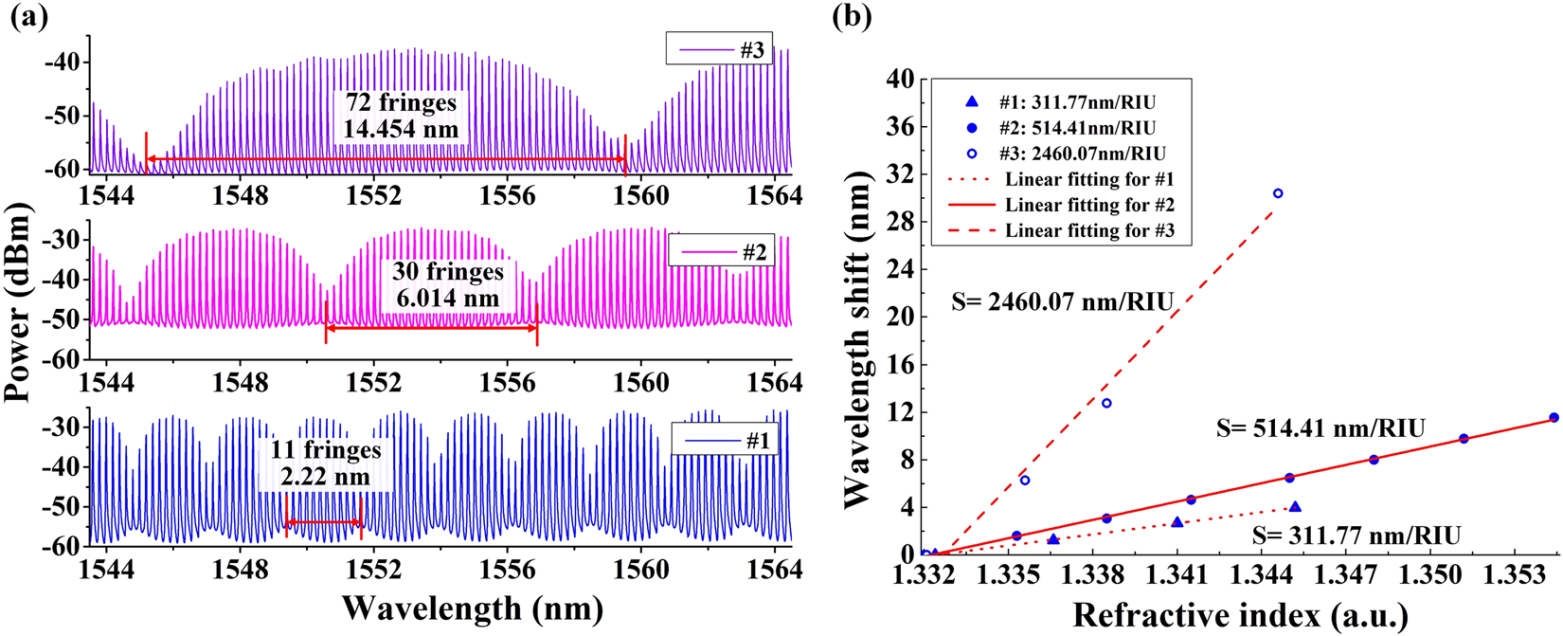
(**a**) Vernier spectra with periods of 2.22 nm, 6.014 nm and 14.454 nm; (**b**) The corresponded relationships between the wavelength shifts of three θ-shaped microfiber resonators and RIs.

In order to demonstrate the controllability of RI sensitivity, we adjusted the cavity length of the fabricated θ-shaped microfiber resonator by manually pulling the microfiber pigtail. In the experiment, we recorded the spectra of the θ-shaped microfiber resonator when its cavity length *L* was tuned to be 9.4 mm, 9 mm and 8.7 mm. The FSRs in the reflection spectra of the θ-shaped microfiber resonator under the three conditions #1, #2 and #3 are calculated as 0.184 nm, 0.194 nm and 0.198 nm, respectively. After cascading with a FFPI with FSR of 0.2 nm, the induced Vernier spectra has different periods of 2.22 nm, 6.01 nm and 14.45 nm, respectively, as shown in Fig. 6(a). The sub-fringes contained in the three Vernier spectra can be counted as 11, 30 and 72, which means their magnification factors *M* are 12, 31 and 73 respectively. The relationships between the wavelength shifts of three microfiber resonators and surrounding RIs are shown in Fig. 6(b). Their RI sensitivities are 311.77 nm/RIU, 514.41 nm/RIU, and 2460.07 nm/RIU separately, indicating that the RI sensitivity of the θ-shaped microfiber resonator can be controlled by adjusting the cavity length.

## Discussions

In the investigation of RI sensitivity controllability, the sensitivities do not strictly follow the ratio of magnification factors *M*, which may result from the un-absolutely uniform microfiber diameter. According to Eq. (5), the deceasing of microfiber diameter will cause the increasing of Δ*n*_*eff*1_/Δ*n*_*a*_^28^ and thus lead the RI sensitivity to be enhanced. In the experiment, limited by the manufacturing platform, the shape of fabricated microfiber is not a cylinder with uniform diameter along the length, but a circular cone with slowly increasing diameter. When pulling the pigtail to adjust cavity length of the θ-shaped microfiber resonator, the microfiber diameter forming the main cavity alters, inducing the sensitivity of the proposed RI sensor to change more or less. In order to conquer this problem, we need to increase the length of microfiber waist, which can be realized by stretching the fiber on both ends and scanning the oxyhydrogen flame over lengths of several tens of mm along the fiber during the fabrication process.

As indicated by Eq. (5), the magnification factor *M* is also related to the effective RI of the modes in the θ-shaped microfiber resonator which is susceptible to surrounding temperature. Therefore, in the future work, we can tune the sensitivity through controlling the temperature around the packaged θ-shaped microfiber resonator, which can be easily implemented by putting it onto a temperature-controllable hotplate.

In summary, we have experimentally demonstrated a reflective θ-shaped microfiber resonator based RI sensor with controllable sensitivity. The θ-shaped microfiber resonator is a specially designed all-fiber structure with comb spectrum under weak coupling condition. By analyzing the mode propagation process, we show that the θ-shaped microfiber resonator could act as a reflective device, which makes it compact, portable, and easily compatible with other fiber devices in practical applications. Benefitting from the large evanescent field of microfiber, the θ-shaped microfiber resonator can perceive the change of surrounding RI, whereas the RI sensitivity is ultra-limited as investigated. In order to enhance the RI sensitivity, we cascade the θ-shaped microfiber resonator with a FFPI to generate Vernier effect. It is because of the Vernier effect and the tunability of the θ-shaped microfiber resonator that the RI sensitivity is enhanced and controllable. Our theoretical analysis and experimental results verify the above expectation. Theoretically, we show that the RI sensitivity of the combined structure with Vernier effect is *m* times higher than the sensitivity of singular θ-shaped microfiber resonator. Moreover, by accommodating cavity length of the θ-shaped microfiber resonator, the magnification factor *M* = (*m* + 1) can be tuned which enables the RI sensitivity to be controled. Experimentally, we verify that the RI sensitivity can be widely tuned from 311.77 nm/RIU to 2460.07 nm/RIU when the cavity length of the θ-shaped microfiber resonator is adjusted from 9.4 mm to 8.7 mm. The θ-shaped microfiber resonator based all-fiber RI sensor featuring controllable sensitivity and compact size can be widely used for chemical and biological detections. The proposed scheme of generating Vernier effect also offers a universal idea to increase measurement sensitivity for other optical fiber sensing structures with comb spectrum.

## Methods

### Fabrication

Fabrication process of the θ-shaped microfiber resonator is outlined in the schematic of Fig. 2(a). Firstly, a microfiber fabricated by flame-heated taper-drawing technique is cut off to form a free microfiber pigtail. Then, after being anchored onto the ridge of a substrate, the free microfiber pigtail is bent and tied twice, forming a θ-shaped cavity. Finally, by slightly adjusting the cavity length and two couplers, a θ-shaped microfiber resonator is completed. In the experiment, by using a microfiber with length of 75 mm and waist diameter of 2 μm, a θ-shaped microfiber resonator with size of 4 *mm* × 2 *mm* is assembled, as presented in the inset of Fig. 2(b). The propagation loss of microfibers is measured as low as 1 dB. Due to the thin diameter and the extremely good flexibility of microfiber, its bending loss can be ignored. In order to keep stable, the fabricated θ-shaped microfiber resonator is placed on a silica substrate coated by Teflon with RI of 1.2924^29^.

### Experimental setup

Experimental setup for RI sensing characterization of the θ-shaped microfiber resonator is displayed in Fig. 2(b). The light launched from a static optical sensing interrogator (Micron Optics, SM125) flows to the θ-shaped microfiber resonator through the port 2 and port 3 of a circulator. Then the reflected light from the θ-shaped microfiber resonator enters the FFPI (*FSR*_2_ = 0.2 *nm*, Micron Optics, FFP-I) through port 4 of the circulator. Finally, the transmitted light from FFPI comes back to the interrogator through port 1 of the circulator and is detected by the interrogator. Measurement and recording of the spectra are conducted in a computer with a bundled software.

### RI sensing experiment

To change the surrounding RI of the θ-shaped microfiber resonator, a batch of glycerin solution with different concentration is tested in the experiment. After each test, we drain out the used glycerin solution, thoroughly clean the substrate on which the θ-shaped microfiber resonator is supported, and then carefully drop new glycerin solution. The corresponding RI of the glycerin solution is calibrated by an Abbe refractometer.
